# Supplementary figures and images for: Exploration of prognostic genes associated with lymphangiogenesis in breast cancer based on transcriptomics and experimental verification
Source: PeerJ. 2025 Aug 27;13:e19890. doi: 10.7717/peerj.19890 (PMC12398285; doi:10.7717/peerj.19890)

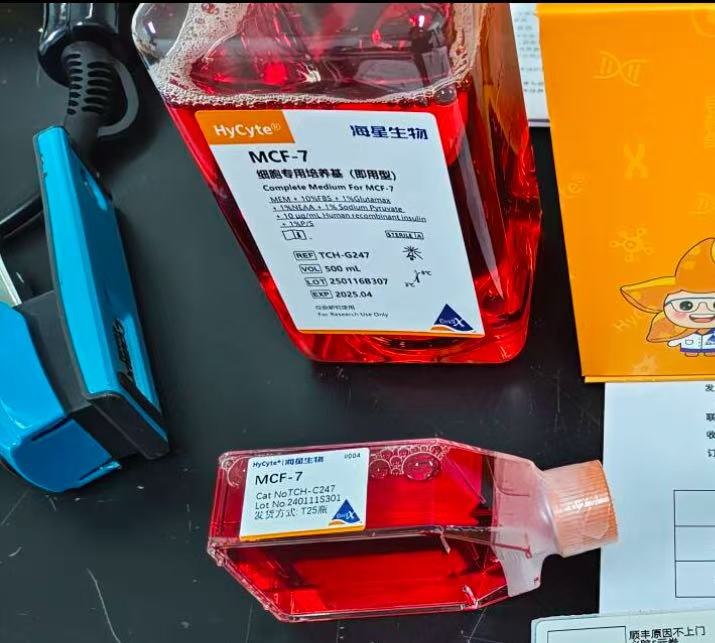

Supplement: Supplemental Information 10 — This dataset contains microscopy images documenting the in vitro culture of breast cancer cell lines (MCF-7, SK-BR-3, ZR-75-1) and normal mammary epithelial cells (MCF-10A). [file peerj-13-19890-s010.zip › Supplemental Data S3/1.MCF-7 cell.png]

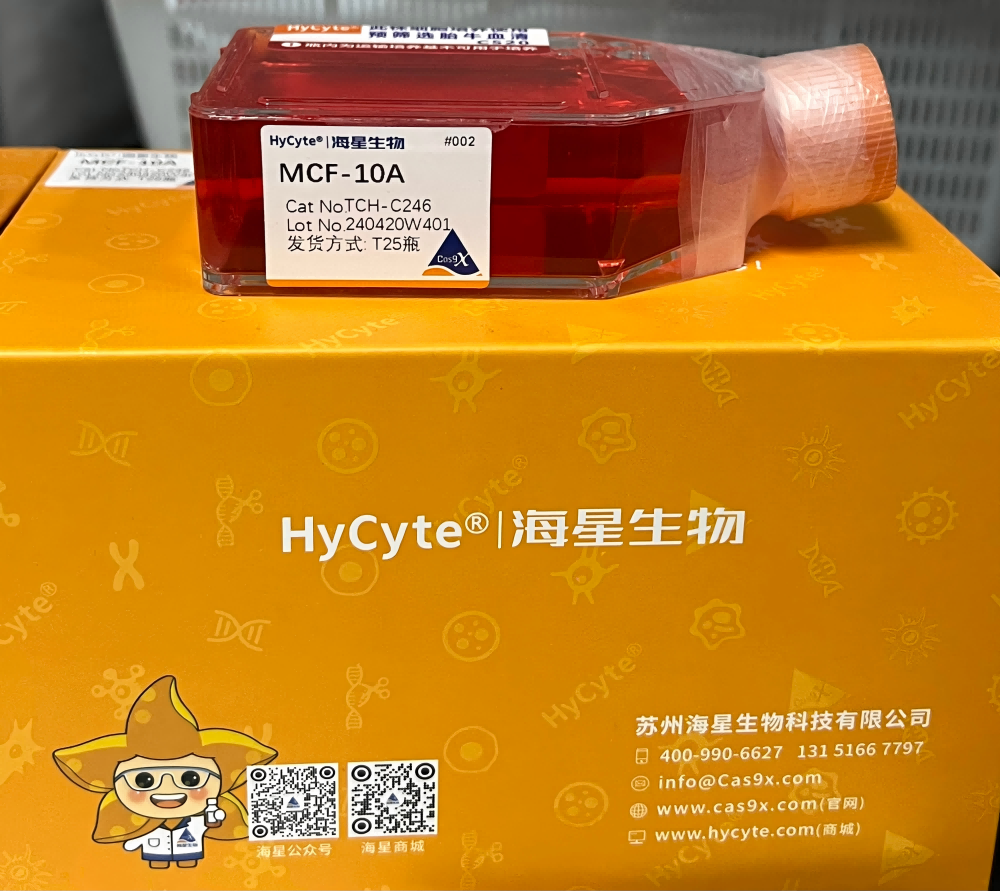

Supplement: Supplemental Information 10 — This dataset contains microscopy images documenting the in vitro culture of breast cancer cell lines (MCF-7, SK-BR-3, ZR-75-1) and normal mammary epithelial cells (MCF-10A). [file peerj-13-19890-s010.zip › Supplemental Data S3/2.MCF-10A cell.png]

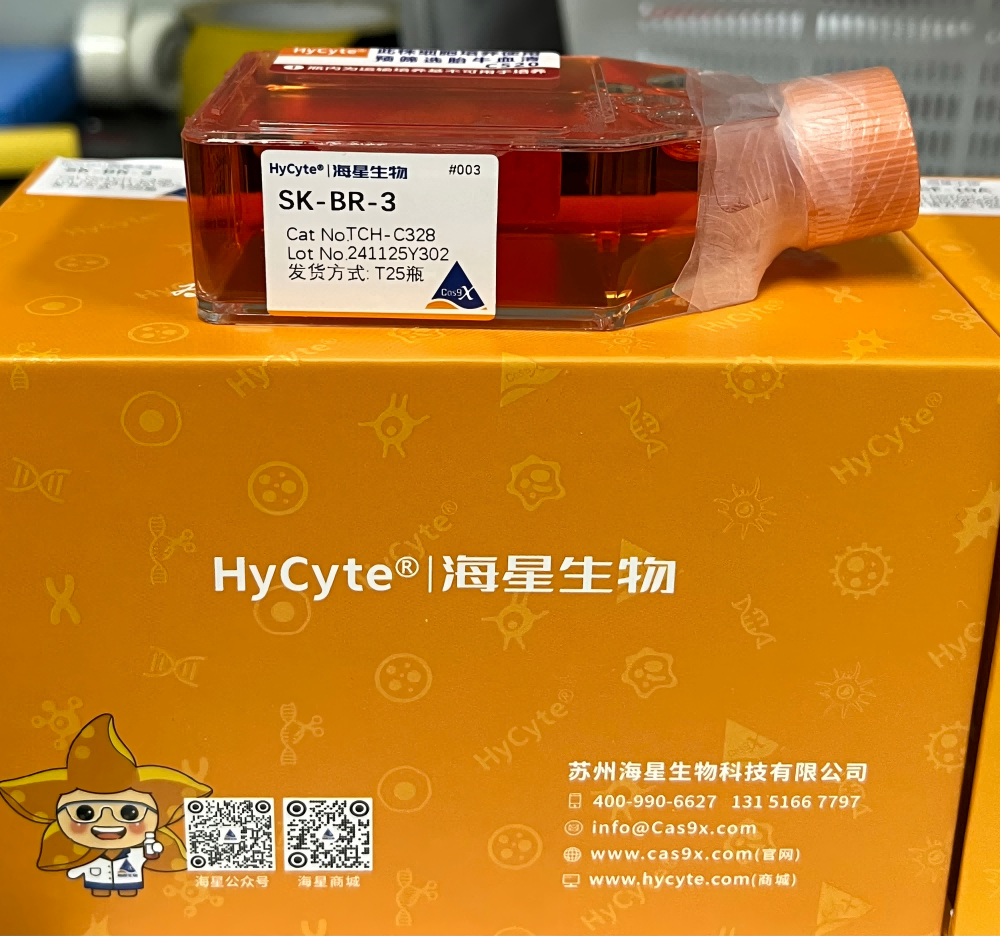

Supplement: Supplemental Information 10 — This dataset contains microscopy images documenting the in vitro culture of breast cancer cell lines (MCF-7, SK-BR-3, ZR-75-1) and normal mammary epithelial cells (MCF-10A). [file peerj-13-19890-s010.zip › Supplemental Data S3/3.SK-BR-3 cell.png]

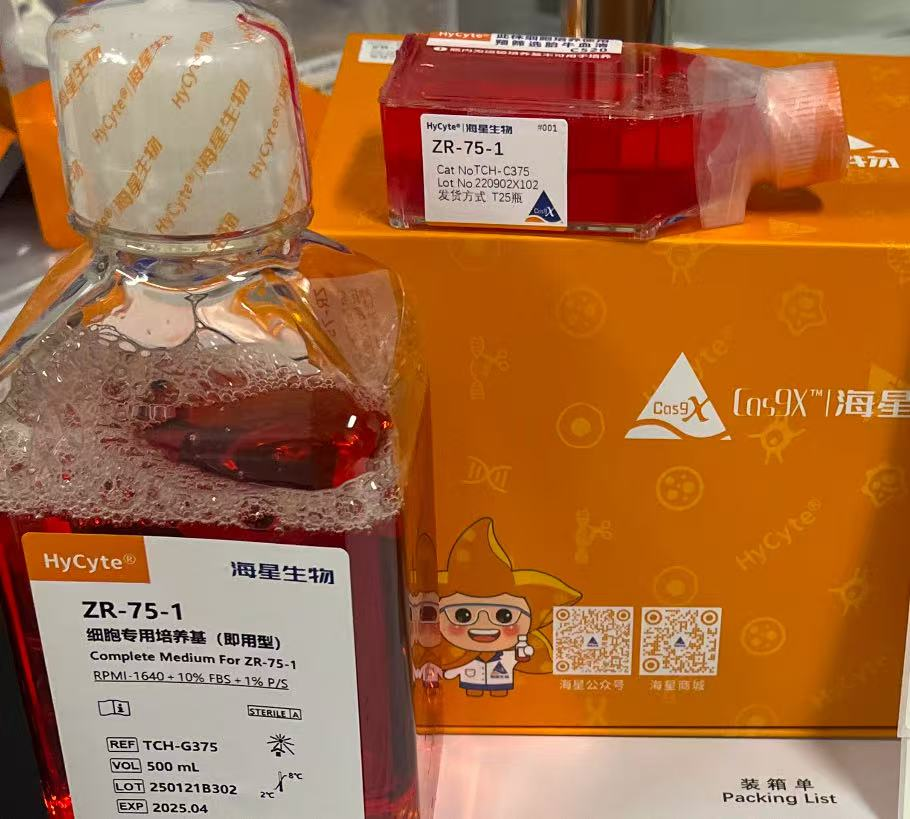

Supplement: Supplemental Information 10 — This dataset contains microscopy images documenting the in vitro culture of breast cancer cell lines (MCF-7, SK-BR-3, ZR-75-1) and normal mammary epithelial cells (MCF-10A). [file peerj-13-19890-s010.zip › Supplemental Data S3/4.ZR-75-1 cell.png]

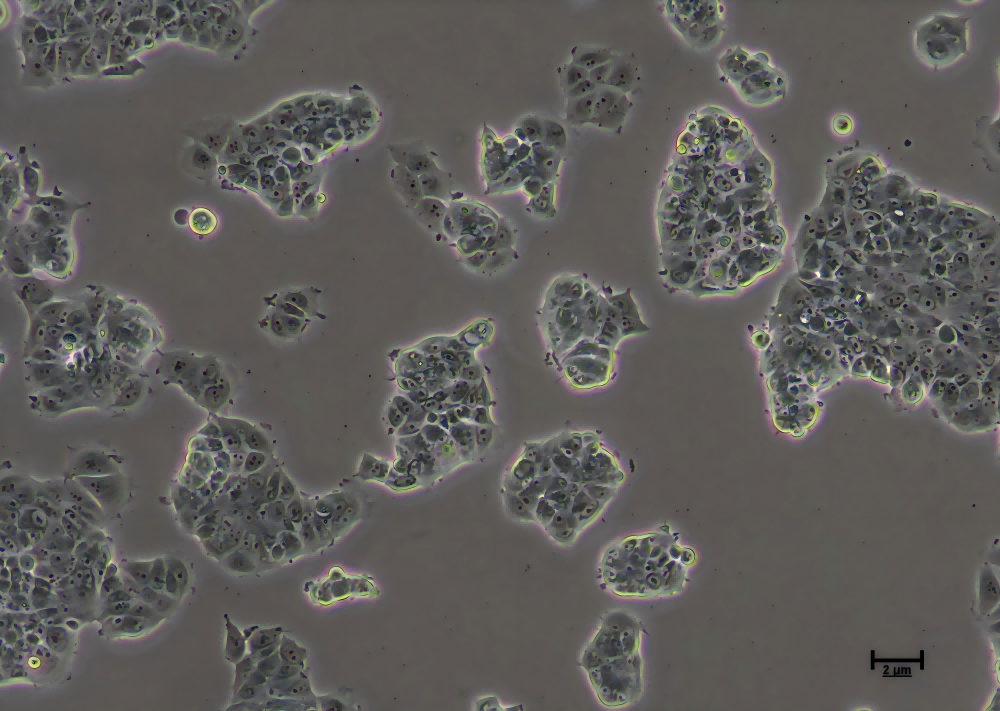

Supplement: Supplemental Information 10 — This dataset contains microscopy images documenting the in vitro culture of breast cancer cell lines (MCF-7, SK-BR-3, ZR-75-1) and normal mammary epithelial cells (MCF-10A). [file peerj-13-19890-s010.zip › Supplemental Data S3/5.MCF-7 cell morphology.png]

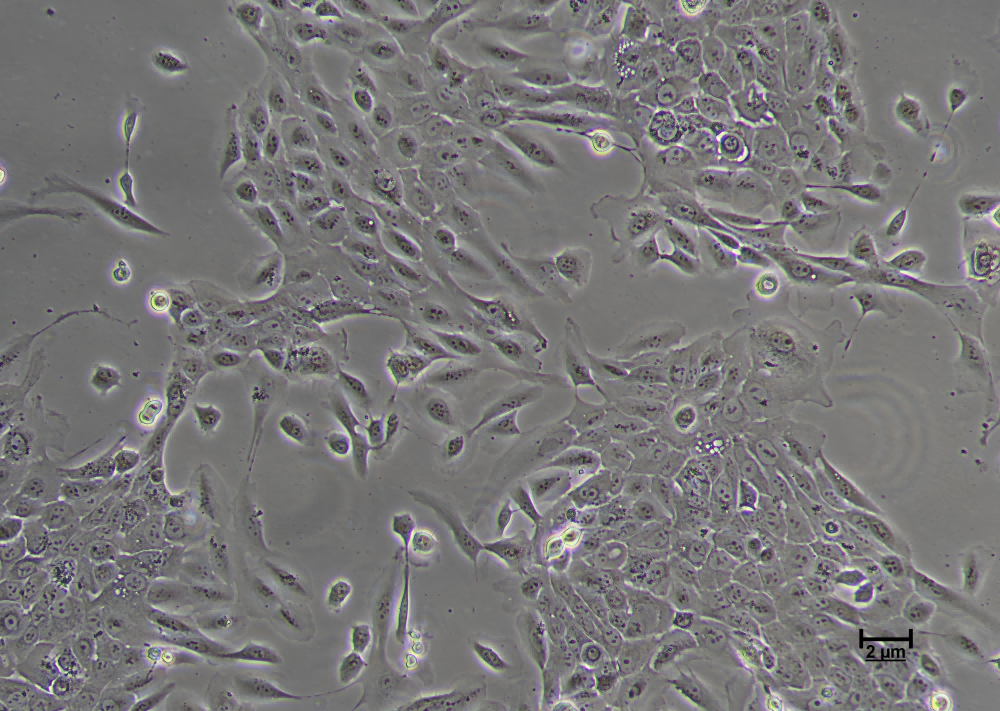

Supplement: Supplemental Information 10 — This dataset contains microscopy images documenting the in vitro culture of breast cancer cell lines (MCF-7, SK-BR-3, ZR-75-1) and normal mammary epithelial cells (MCF-10A). [file peerj-13-19890-s010.zip › Supplemental Data S3/6. MCF-10A cell morphology.png]

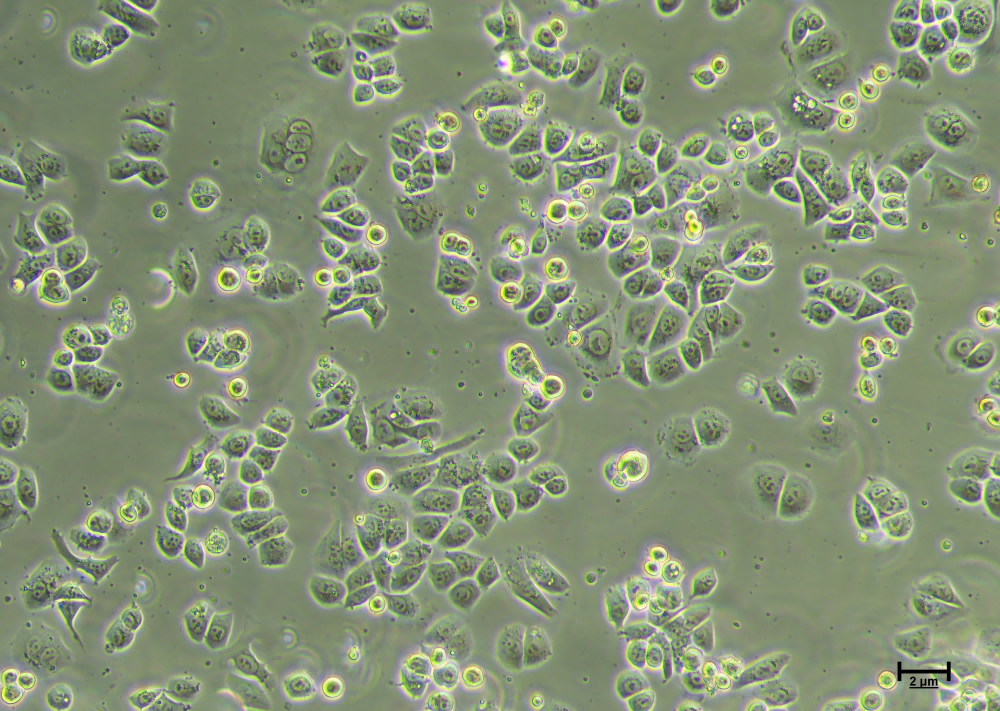

Supplement: Supplemental Information 10 — This dataset contains microscopy images documenting the in vitro culture of breast cancer cell lines (MCF-7, SK-BR-3, ZR-75-1) and normal mammary epithelial cells (MCF-10A). [file peerj-13-19890-s010.zip › Supplemental Data S3/7. SK-BR-3 cell morphology.png]

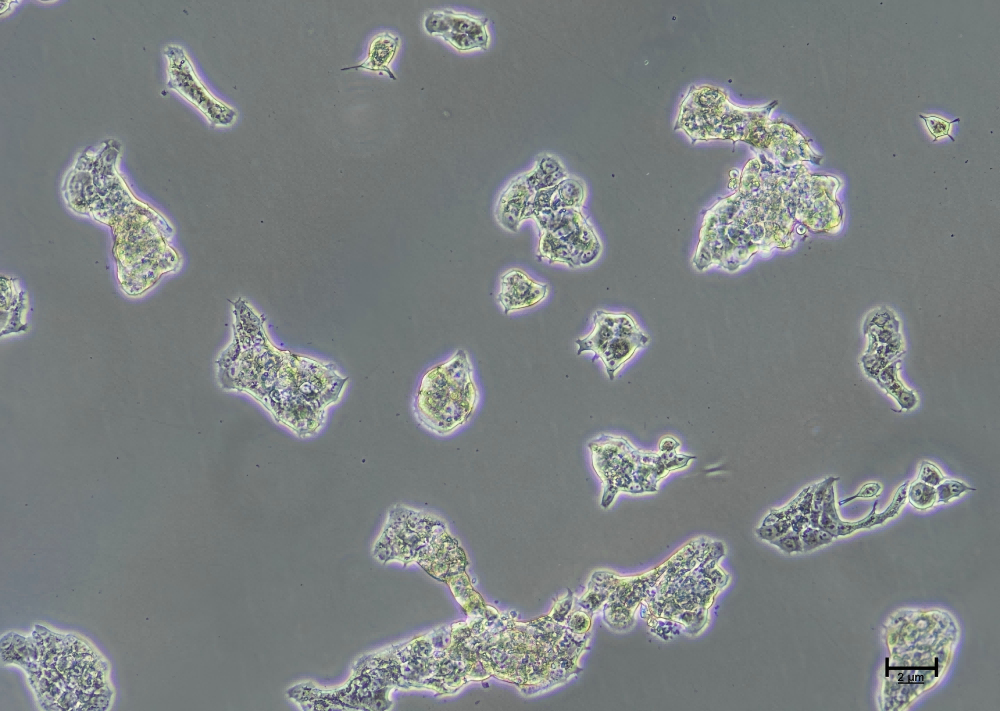

Supplement: Supplemental Information 10 — This dataset contains microscopy images documenting the in vitro culture of breast cancer cell lines (MCF-7, SK-BR-3, ZR-75-1) and normal mammary epithelial cells (MCF-10A). [file peerj-13-19890-s010.zip › Supplemental Data S3/8.ZR-75-1 cell morphology.png]

**Relative CCL19 level  
(to GAPDH)**

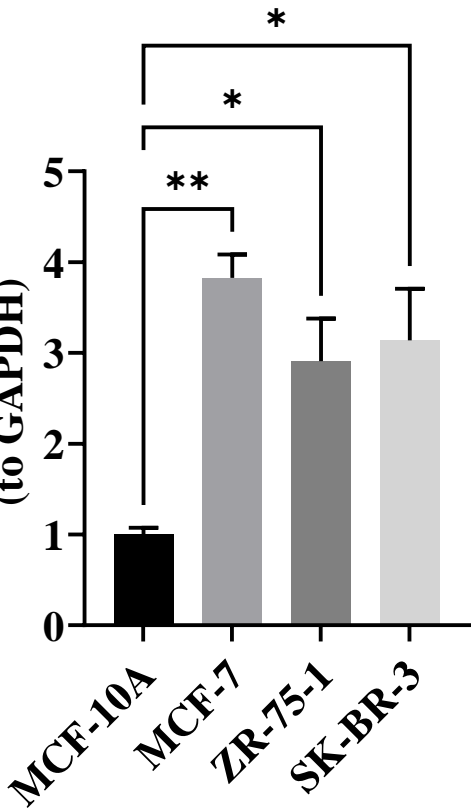

Supplement: Supplemental Information 11 — ΔΔCt values from triplicate qPCR assays. Cell lines: MCF-10A (normal), MCF-7, ZR-75-1,SK-BR-3 (cancer). Targets: CCL19, CD24, CEBPD, ZIC2. Reference: GAPDH. Data columns: [Sample, Ct, ΔCt, ΔΔCt, FoldChange(2−ΔΔCt)]. [file peerj-13-19890-s011.zip › Supplemental Data S4/4.Histogram/CCL19.pdf]

**Relative CD24 level  
(to GAPDH)**

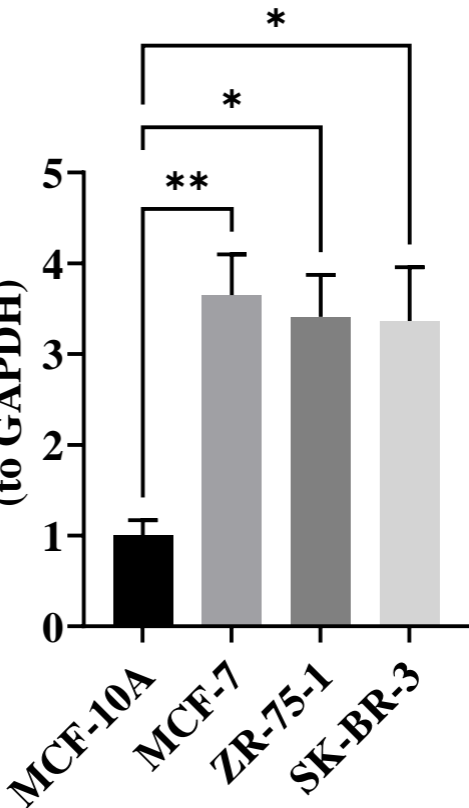

Supplement: Supplemental Information 11 — ΔΔCt values from triplicate qPCR assays. Cell lines: MCF-10A (normal), MCF-7, ZR-75-1,SK-BR-3 (cancer). Targets: CCL19, CD24, CEBPD, ZIC2. Reference: GAPDH. Data columns: [Sample, Ct, ΔCt, ΔΔCt, FoldChange(2−ΔΔCt)]. [file peerj-13-19890-s011.zip › Supplemental Data S4/4.Histogram/CD24.pdf]

**Relative CEBPD level  
(to GAPDH)**

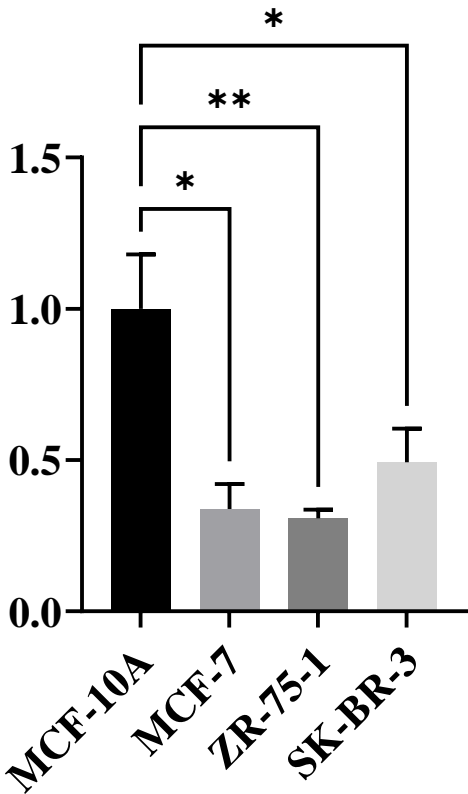

Supplement: Supplemental Information 11 — ΔΔCt values from triplicate qPCR assays. Cell lines: MCF-10A (normal), MCF-7, ZR-75-1,SK-BR-3 (cancer). Targets: CCL19, CD24, CEBPD, ZIC2. Reference: GAPDH. Data columns: [Sample, Ct, ΔCt, ΔΔCt, FoldChange(2−ΔΔCt)]. [file peerj-13-19890-s011.zip › Supplemental Data S4/4.Histogram/CEBPD.pdf]

**Relative ZIC2 level  
(to GAPDH)**

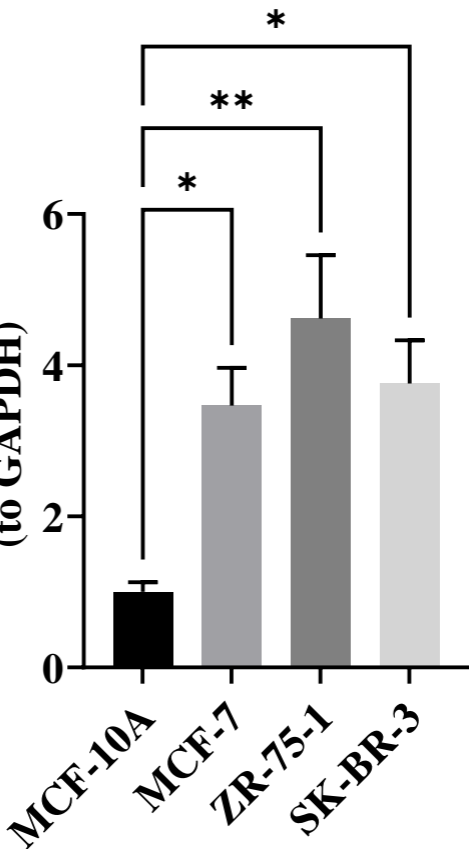

Supplement: Supplemental Information 11 — ΔΔCt values from triplicate qPCR assays. Cell lines: MCF-10A (normal), MCF-7, ZR-75-1,SK-BR-3 (cancer). Targets: CCL19, CD24, CEBPD, ZIC2. Reference: GAPDH. Data columns: [Sample, Ct, ΔCt, ΔΔCt, FoldChange(2−ΔΔCt)]. [file peerj-13-19890-s011.zip › Supplemental Data S4/4.Histogram/ZIC2.pdf]
